# A System of Human Anatomy, General and Special

**Published:** 1844-10

**Authors:** 


ART. V.—*A System of Human Anatomy, General and Special.* By ERASMUS WILSON, M.D., Lecturer on Anatomy, London. Second American edition, edited by PAUL B. GODDARD, A.M., M.D., &c. &c. With over 200 illustrations by Gilbert. From the second London edition. Philadelphia: Lea & Blanchard. 1844. 8vo. pp. 607.

The author of this very popular work has the following words in the preface to his second edition: "I have endeavored to render the present edition more perfect than the preceding, by entering more fully into the description of such parts as were only scantily treated in the first. I regret that these additions have increased the size of the volume,—an effect that it has always been my foremost desire to avoid; for if a large and a verbose book be at any time a great evil, it is so to its fullest extent in a volume which is intended to record only facts, as is the case with a work on Anatomy." The editor in his preface to the second American edition says:

"A chapter on Histology has been prefixed (to this edition) and a considerable number of new cuts added. Among the latter are included some very fine ones of the nerves, which were almost wholly omitted from the original work."

In these sentences we have a brief account of the additions which have been made to the second edition of Wilson's Anatomy, the merits of which work are fully appreciated by the profession, as is evinced by the fact that a second edition has been called for in the short space of two years. The point in which this *vade mecum* stands almost unrivalled, is the beauty of its plates, and whole mechanical execution. As a work of art, it ranks in the very first class of American productions in that department. Its paper, type and engravings are of correspondent excellence, and are such as to do ample justice to the subject matter of the volume, which, it strikes us, is presented in a clear, concise style suited to the character of such a work. Y.

